# Comment on “Attitudes and Health Behavior of Lawyers in Coimbatore, Tamil Nadu”

**DOI:** 10.1155/2016/7836020

**Published:** 2016-01-26

**Authors:** Siddharudha Shivalli

**Affiliations:** Department of Community Medicine, Yenepoya Medical College, Yenepoya University, Mangalore, Karnataka 575018, India

I read the article titled “Attitudes and Health Behavior of Lawyers in Coimbatore, Tamil Nadu” by Barani and Sabapathy [1] with curiosity. The authors' efforts are admirable. This study provides an overview and genderwise differences in attitudes and practices of lifestyle, food intake, and alcohol and tobacco intake among lawyers in Coimbatore, Tamil Nadu. However, the following issues and concerns need to be addressed.

In Materials and Methods, the authors state that a descriptive cross-sectional study was conducted on 100 lawyers selected by convenience sampling. However, the authors have not mentioned the ethical clearance for this study and informed consent from the participants, for voluntary participation.

It is highly recommended to follow the STROBE (strengthening the reporting of observational studies in epidemiology) checklist while reporting findings of a cross-sectional study [2]. It is endorsed by a number of reputed biomedical journals. According to STROBE checklist, the study should have predecided inclusion and exclusion criteria for the study participants. In addition, outcome variables in observational study should be reported with their precision (e.g., 95% confidence interval) [2].

Use of the chi-square tests is inappropriate, if any expected frequency is <1 or if the expected frequency is <5 in >20% of the cells. In Table 2 of the study by Barani and Sabapathy [1], chi-square calculations for “BMI” and “type of work” show an expected count of <5 in >20% of the cells. The authors could have either clubbed the subgroups logically and hence reduced the degrees of freedom or used apt correction for chi-square. In our opinion, rowwise percentage calculation, rather than columnwise, would have eased the interpretation of results.

According to STROBE guidelines, the authors should discuss limitations of the study, taking into account sources of potential bias or imprecision [2]. Findings of this study may not be applicable to other lawyers in Coimbatore, Tamil Nadu, India, as the study was done on 100 lawyers selected by convenience sampling.

Nonetheless, we must applaud the authors for investigating an important health-related issue among lawyers.
